# Kruppel-Like Factor 4 Positively Regulates Autoimmune Arthritis in Mouse Models and Rheumatoid Arthritis in Patients *via* Modulating Cell Survival and Inflammation Factors of Fibroblast-Like Synoviocyte

**DOI:** 10.3389/fimmu.2018.01339

**Published:** 2018-06-27

**Authors:** Seungjin Choi, Kijun Lee, Hyerin Jung, Narae Park, Jaewoo Kang, Ki-Hoan Nam, Eun-Kyeong Kim, Ji Hyeon Ju, Kwi Young Kang

**Affiliations:** ^1^CiSTEM Laboratory, Catholic iPSC Research Center, College of Medicine, The Catholic University of Korea, Seoul, South Korea; ^2^Division of Rheumatology, Department of Internal Medicine, Seoul St. Mary’s Hospital, College of Medicine, The Catholic University of Korea, Seoul, South Korea; ^3^School of Biological Sciences, Institute of Molecular Biology and Genetics, Seoul National University, Seoul, South Korea; ^4^Department of Cancer Biomedical Science, Research Institute, National Cancer Center, Goyang, South Korea; ^5^Laboratory Animal Resource Center, Korea Research Institute of Bioscience & Biotechnology (KRIBB), Cheongju, South Korea; ^6^Division of Rheumatology, Department of Internal Medicine, Incheon St. Mary’s Hospital, The Catholic University of Korea, Incheon, South Korea

**Keywords:** Kruppel-like factor 4, rheumatoid arthritis, fibroblast-like synoviocyte, inflammation, matrix metalloproteinase, collagen-induced arthritis, collagen antibody-induced arthritis

## Abstract

Rheumatoid arthritis (RA) is a chronic autoimmune disease that causes mild to severe joint inflammation. During RA pathogenesis, fibroblast-like synoviocytes (FLS) acquire a tumor-like phenotype and mediate cartilage destruction both directly and indirectly by producing proinflammatory cytokines and matrix metalloproteinases (MMPs). Kruppel-like factor (KLF) 4, a member of the KLF family, plays significant roles in cell survival, proliferation, and differentiation. A recent study reported increased expression of KLF4 in synovial tissue from RA patients. However, its precise role in RA in different models, including mouse autoimmune disease models, remains unclear. In this study, we examined the role of KLF4 during development of autoimmune arthritis in mouse models. To do this, we used KLF4 knockout mice rendered by ribonucleic acid (RNA)-guided endonuclease (RGEN) and performed collagen antibody-induced arthritis (CAIA). We found that deletion of KLF4 reduces inflammation induced by CAIA. In addition, we assessed collagen-induced arthritis (CIA) in control mice and KLF4-overexpressing mice generated by a minicircle vector treatment. Severity of CIA in mice overexpressing KLF4 was greater than that in mice injected with control vector. Finally, we verified the inflammatory roles of KLF4 in CIA by treating Kenpaullone which is used as KLF4 inhibitor. Next, we focused on human/mouse FLS to discover the cellular process involved in RA pathogenesis including proliferation, apoptosis, and inflammation including MMPs. In FLS, KLF4 upregulated expression of mRNA encoding proinflammatory cytokines interleukin (IL)-1β and IL-6. KLF4 also regulated expression of matrix metallopeptidase 13 in the synovium. We found that blockade of KLF4 in FLS increased apoptosis and suppressed proliferation followed by downregulation of antiapoptotic factor BCL2. Our results indicate that KLF4 plays a crucial role in pathogenesis of inflammatory arthritis *in vivo*, by regulating apoptosis, MMP expression, and cytokine expression by FLS. Thus, KLF4 might be a novel transcription factor for generating RA by modulating cellular process of FLS.

## Introduction

Kruppel-like factors (KLFs) are transcription factors that share homology with the *Drosophila* gene Kruppel. These proteins are characterized by three zinc finger motifs located at the carboxy terminus. The N terminus of KLFs mediates activation or repression of transcription and other protein-protein interactions (1, 2). KLF4, a member of the KLF family, plays significant roles in stem cell function, cell survival, proliferation, and differentiation (3–5). KLF4 was originally identified as a gut-enriched transcription factor in epithelial cells lining the gut (6). It plays crucial roles not only in terminal differentiation and growth of epithelial cells in the gut and skin but also in regulating diverse cellular processes (7, 8). Recently, KLF4 has gained attention as one of four factors that induce pluripotent stem cells (9).

Kruppel-like factor 4 is also implicated in regulation of inflammation. Its role in inflammation came to light in several reports showing that in macrophages it interacts physically with TNF-α and the NF-κB p65 subunit to induce the NOS2 promoter in response to interferon-γ (IFN-γ) and lipopolysaccharide (LPS) (2). Also, KLF4 binds on late inflammatory cytokine promoter, and promotes its expression, translocation, and release in RAW 264.7 macrophages in response to LPS stimulation (10). In addition, KLF4 plays a role in secretion of inflammatory cytokines by monocytes (3, 4) and dendritic cells (11). Inflammatory molecules IFN-γ and LPS are required by KLF4 to target inflammatory genes in mature cells (2). These results suggest the importance role of KLF4 for the development of inflammatory responses. A recent study showed that KLF4 promotes acute colitis in an animal model of inflammatory bowel disease, and that deletion of KLF4 from the intestinal epithelium ameliorates chemically induced colitis (12). This suggests that KLF4 may be a therapeutic target in the context of inflammatory disease.

Rheumatoid arthritis (RA) is a chronic autoimmune disease that causes mild to severe joint inflammation. The pathogenesis of RA is complex and involves many cell types, including T cells, B cells, macrophages, and fibroblast-like synoviocytes (FLS) (13). RA is characterized by tumor-like hyperplasia of the synovial membrane, which is caused by an increase in the number of FLS and infiltration and accumulation of inflammatory cells (13). FLS not only form a cellular mesh that facilitates the inflammatory process but also exhibit a tumor cell-like transformation. During RA pathogenesis, FLS acquire a tumor-like phenotype and mediate cartilage destruction both directly and indirectly by producing proinflammatory cytokines and matrix metalloproteinases (MMPs) (14). Targeting the tumor-like hyperplasia characteristics of FLS might improve the clinical outcome of inflammatory arthritis without suppressing systemic immunity. A recent study reported increased expression of KLF4 in synovial tissue from RA patients. *In vitro*, KLF4 regulates expression of the proinflammatory cytokine interleukin (IL)-6 in FLS isolated from RA patients and induced by TNF-α (15). KLF4 is involved in the inflammatory response and in expression of proinflammatory cytokines by FLS; thus KLF4 may regulate RA-associated inflammation. However, no study has examined its role in development of inflammatory arthritis in mouse models of RA. In addition, its role in activated FLS is unclear.

Since KLF4 plays important roles in activation of inflammatory cells and FLS, the aim here was to examine expression of KLF4 during development of inflammatory arthritis in mouse models of RA, a disease characterized by infiltration of joints by inflammatory cells and hyperplasia of the synovium. We examined the role of KLF4 during development of inflammatory arthritis in KLF4 knockout mice and minicircle-treated KLF4-overexpressing mice with inflammatory arthritis. We also examined the underlying mechanism(s), focusing on the role of inflammation factors and MMPs secreted by FLS.

## Materials and Methods

### Mice

All mice were maintained under specific pathogen-free conditions. The mice were housed in polycarbonate cages and fed standard mouse chow (Ralston Purina) and water. All mouse protocols were approved by the institutional Animal Care and Use Committee of the School of Medicine, the Catholic University of Korea. Ribonucleic acid (RNA)-guided endonuclease (RGEN)-mediated KLF4 knockout mice on a C57BL/6 background (KLF4^Rg−/−^) were generated at the Korea Research Institute of Bioscience & Biotechnology. As male mice are reported to have greater susceptibility to CAIA than female mice (16), only male mice aged 8–12 weeks were used as models of collagen antibody-induced arthritis (CAIA). Six-week-old DBA1/J female mice were purchased from Orient Co. Ltd. (Sungnam, South Korea) and used as models of collagen-induced arthritis (CIA) within 2 weeks. All experiments were approved by the Animal Research Ethics Committee at the Catholic University of Korea.

### Isolation and Culture of FLS

Human synoviums were obtained from three RA patients and three OA patients for non-RA controls undergoing knee surgery. All patients satisfied the 1987 revised criteria of the American College of Rheumatology (17). Tissue samples were minced into pieces (1–3 mm) and incubated for 4 h (at 37°C/5% CO_2_) with 4 mg/ml crude collagenase (Merck, Darmstadt, Germany) in Dulbecco’s Modified Eagle Medium (Gibco, MA, USA). Dissociated cells were centrifuged at 500 *g* and resuspended in DMEM containing 20% fetal bovine serum (FBS, Gibco). After 12–24 h, non-adherent cells were removed, and remaining adherent cells were cultured in DMEM supplemented with 20% FBS. The cultures were maintained at 37°C/5% CO_2_ for 14 days. FLS from passages 3 to 8 were used for subsequent experiments. All patients provided informed consent, and the study was approved by the Institutional Review Board of Incheon St. Mary’s hospital (license no. OC17TNSI0155). Murine FLS were isolated from hip joint tissue from C57BL/6 mice as previously described (18).

### Induction of CAIA

Collagen antibody-induced arthritis was induced in KLF4^Rg−/−^ and KLF4^+/+^ (wild-type) mice by intraperitoneal (i.p.) injection of 5 mg of a 5-clone collagen antibody cocktail (Chondrex, Redmond, WA, USA). After 3 days, mice were injected i.p. with 50 µg of LPS. Severity of arthritis was evaluated clinically as described previously (19). Hind paw thickness was measured with a thickness ruler.

### Induction of CIA and Treatment

Bovine type II collagen (CII; Chondrex) was dissolved in 0.1 M acetic acid (final concentration, 2 mg/ml). CII was emulsified equally with complete Freund’s adjuvant (CFA; Chondrex) containing 2 mg/ml heat-killed *Mycobacterium tuberculosis*. Female DBA1/J mice were injected intradermally with 0.1 ml of the CII/CFA emulsion on day 0. Mice then received intradermally with 0.1 ml of 2 mg/ml CII emulsified in incomplete Freund’s adjuvant (IFA; Chondrex) (2 mg/ml) on day 21 [for mice treated with Kenpaullone (a KLF4 inhibitor; Cayman Chemical, Ann Arbor, MI, USA)] and day 28 (for mice transduced with the minicircle vector). Injection of CII/IFA into minicircle-transduced mice took place 7 days later than that into Kenpaullone-treated mice to induce lower activity of arthritis. Severity of arthritis was scored as previously described (19).

### Isolation of RNA, Synthesis of Complementary Deoxyribonucleic Acid (cDNA), and Real-Time Quantitative Polymerase Chain Reaction (q-PCR)

Isolated cells were treated with Trizol reagent, mixed with 200 µl chloroform, and centrifuged at 8,000 *g* at 4°C. The supernatant was recovered, mixed with 500 µl 2-propanol, and centrifuged at 8,000 *g* at 4°C. The mRNA pellet was extracted with 75% ethanol to increase RNA purity. After centrifugation, ethanol was removed by evaporation, and the pellet was dissolved in diethylpyrocarbonate-treated distilled water (Tech & Innovation, Chuncheon, South Korea). A reverse-transcription aid kit (Thermo Scientific, MA, USA) was used to synthesize cDNA, which was then subjected to q-PCR using SYBR Green PCR Master Mix (Roche, Basel, Switzerland) and a LightCycler 480 Instrument II (Roche). GAPDH was used as an internal control. The sequences of all primers used for PCR are listed in Table 1.

**Table 1:** Primers used for real-time PCR.

Species	Target name	Direction	Primer sequence
Mouse	GAPDH	Forward	ACCCCAGCAAGGACACTGAGCAAG
		Reverse	TGGGGGTCTGGGATGGAAATTGTG
	Kruppel-like factor (KLF) 4	Forward	CCAGACCAGATGCAGTCACAA
		Reverse	ACGACCTTCTTCCCCTCTTTG
	Interleukin (IL)-6	Forward	CACGGCCTTCCCTACTTCAC
		Reverse	CTGCAAGTGCATCATCGTTGT
	IL-1β	Forward	ATGCCACCTTTTGACAGTGATG
		Reverse	AGCTTCTCCACAGCCACAAT
	Matrix metalloproteinase (MMP) 3	Forward	GGGAAGCTGGACTCCAACAC
		Reverse	GCGAACCTGGGAAGGTACTG
	MMP9	Forward	CACGGTTGGCCCTACAGGCG
		Reverse	AGGCCTCAGAAGAGCCCGCA
	MMP13	Forward	GCACTGCTGGGCACCATGCAT
		Reverse	GGGAAGGGGCAGGGACCAACA
	TNF-α	Forward	CCTCACACTCACAAACCACCA
		Reverse	GTGAGGAGCACGTAGTCGG
	BCL2	Forward	CCGGGAGAACAGGGTATGAT
		Reverse	GCACAGCGGGCATTGGGTTG
	IL-17A	Forward	TCTCCACCGCAATGAAGACC
		Reverse	CACACCCACCAGCATCTTCT

Human	GAPDH	Forward	ACCCACTCCTCCACCTTTGA
		Reverse	CTGTTGCTGTAGCCAAATTCGT
	KLF4	Forward	TTCCCATCTCAAGGCACAC
		Reverse	GGTCGCATTTTTGGCACT
	BCL2	Forward	CCAATTGTGCCGAGAAAAGC
		Reverse	GACAGAGCCAGTATTGGGAGTTG
	IL-6	Forward	GGTACATCCTCGACGGCATCT
		Reverse	GTGCCTCTTTGCTGCTTTCAC
	MMP2	Forward	ACACCAAGAACTTCCGTCTG
		Reverse	TGCAGATCTCAGGAGTGACA
	MMP12	Forward	GATCCAAAGGCCGTAATGTT
		Reverse	GGTTCTCTTTTGGGTCTCCA
	MMP13	Forward	CTGCATATGAGCACCCTTCT
		Reverse	TTTTGGAAGACCCAGTTCAG
	Interferon-γ	Forward	CCAACGCAAAGCAATACATGA
		Reverse	CCTTTTTCGCTTCCCTGTTTTA

### Histologic Analysis

Mouse hindlimbs (HLs) were removed, fixed overnight in 4% paraformaldehyde, incubated for 3 weeks in 5% EDTA decalcification buffer, embedded in paraffin, and sliced into 4 µm sections. For immunohistochemistry (IHC), sections were stained with hematoxylin and eosin (H&E) to evaluate immune cell infiltration and pannus formation. Sections were also stained with Safranin O and Toluidine blue to examine cartilage destruction. Anti-vimentin (Abcam, Cambridge, UK), anti-CD90 (Bio-Rad, Hercules, CA, USA), anti-KLF4 (Abcam) antibodies and DAPI (4′,6-diamidino-2-phenylindole) were used for IHC. Sections were examined under an immunofluorescence microscope (Leica, Wetzlar, Germany).

### *In Vitro* Th17 Cell Differentiation

CD4^+^ T cells were isolated from mouse splenocytes (SPL) using anti-mouse CD4 microbeads (Miltenyi Biotec, Bergisch Gladbach, Germany). The sorted CD4^+^ T cells were stimulated with plate-bound anti-mouse CD3 (BD Biosciences, CA, USA) and anti-mouse CD28 (BD) and cultured with anti-mouse IFN-γ (R&D Systems, MN, USA) and IL-4 (R&D Systems) to block Th1 and Th2 differentiation, respectively. Th17 differentiation was induced by culture with recombinant IL-6 (R&D Systems) and recombinant transforming growth factor-β1 (R&D Systems) for 3 days.

### Lentiviral Transduction

piLenti-small-interfering RNA-GFP-based scrambled (si-scr) sequence-integrated vector was used as control, and piLenti-si-KLF4-GFP (si-KLF4) sequence-integrated vector (abm, Vancouver, BC, Canada) was used to knock down KLF4. HEK293T cells were transfected with si-scr and si-KLF4 using Lipofectamine 2000 Reagent (Thermo Scientific). The virus-containing supernatant was collected and used to transduce human FLS cells (2 × 10^4^ cells/well) as previously described (20).

### Assessment of Cell Viability and Death

All viability assays were performed using Cell Counting Kit-8 (CCK-8, Dojindo, MD, USA). Murine/human FLS were seeded in a 96-well plate at a density of 1 × 10^3^ cells/well. Cells were incubated with LPS (500 ng/ml) for 24 h, followed by addition of 10 µl of CCK-8 reagent to each well. After 4 h, absorbance was measured at 450 nm in a microplate reader (Molecular Devices, CA, USA). All apoptosis assays were based on Annexin V apoptosis staining kit (BD) and flow cytometry analysis (BD).

### Flow Cytometry Analysis of Th17 Cells

Th17-differentiated CD4^+^ T cells were stained with anti-CD4-APC (eBioscience, CA, USA). Cells were then permeabilized in flow cytometry fixation and permeabilization buffer (eBioscience) and stained with an anti-RORγT-PE (eBioscience). Flow cytometry analysis was performed using a BD Fluorescence Activated Cell Sorter Canto I (BD). Data were analyzed using FlowJo (BD).

### Immunoblot Analysis

Harvested FLS were directly lysed by RIPA buffer. Protein lysates were loaded onto SDS-PAGE gels, transferred to a PVDF membrane (Millipore, Darmstadt, Germany), and probed with an anti-KLF4 (1:500, Abcam) antibody, followed by incubation with peroxidase-conjugated Affinipure Goat Anti-Rabbit IgG (Jackson Immune Research, PA, USA). The horseradish peroxidase signal was generated using a western blot detection kit (Abfrontier, Seoul, Korea) and detected using a chemiluminescence system (GE, MA, USA).

### Collagen Degradation Assay

Human RA FLS transduced with si-scr and si-KLF4 were stimulated (or not) with LPS for 24 h and then subjected to a collagen degradation assay as previously described (21). Absorbance was measured at 570 nm in a microplate reader (Molecular Devices).

### Statistical Analysis

Data are expressed as the mean ± SEM. A *t*-test was used to analyze non-parametric quantitative datasets, and a one-tailed *p* value was calculated. Statistical analysis was performed using GraphPad Prism 5 software. *P* < 0.05 was considered statistically significant.

## Results

### KLF4 Is Highly Expressed in Synoviocytes From CIA Mouse

**Figure 1:**
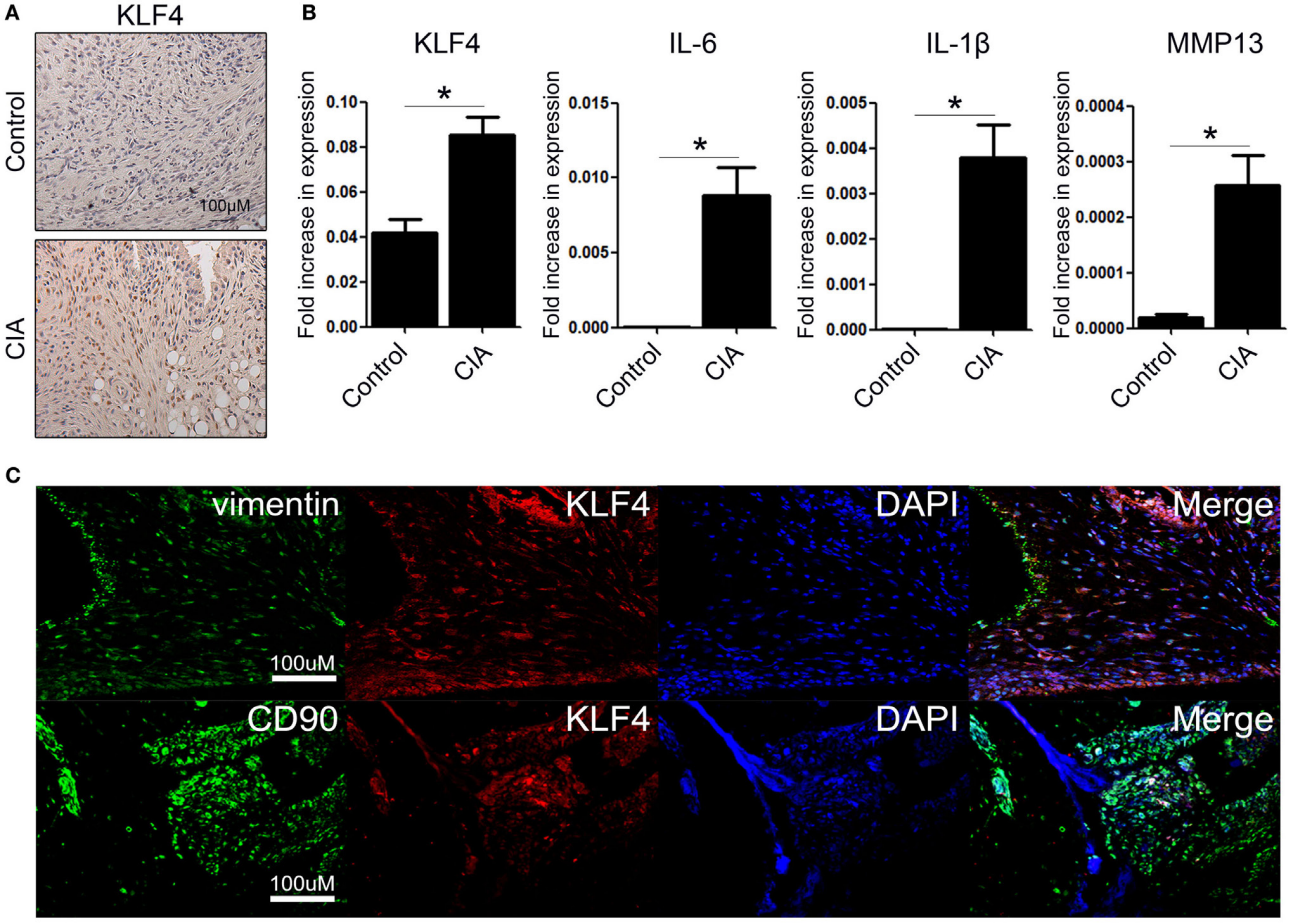
Kruppel-like factor (KLF) 4 is upregulated in joint tissue from collagen-induced arthritis (CIA) mouse. **(A)** Representative immunohistochemistry images of joint tissue sections from control and CIA mice stained with an anti-KLF4 antibody (red). Original magnification, 200×. **(B)** Expression of mRNA encoding KLF4, interleukin (IL)-6, IL-1β, and matrix metalloproteinase (MMP) 13 in murine fibroblast-like synoviocytes in response to lipopolysaccharide (LPS) (as measured by qRT-PCR). The concentration of LPS in the medium was 500 ng/ml. **(C)** Representative immunofluorescence assay images of tissue sections stained with anti-vimentin, anti-CD90, anti-KLF4 antibodies and DAPI (4′,6-diamidino-2-phenylindole). Original magnification, 200×. Values in panel **(B)** are expressed as the mean ± SEM (*P < 0.05).

To examine KLF4 expression in the CIA mouse model, DBA1/J mice were treated with bovine CII mixed with CFA. At 8 weeks after the second immunization, control and CIA mice were sacrificed and joint tissues and paws were harvested. Expression of KLF4 mRNA and protein was upregulated in joint tissue from CIA mice; inflammation status was verified by increased expression of IL-6, IL-1β, and matrix metallopeptidase 13 (MMP13) (Figures 1A,B). To identify the KLF4-expressing cell population, joint tissue was stained with antibodies that will bind to FLS (vimentin, CD90), KLF4, and cell nuclei. Tissues were then examined by immunofluorescence analysis. The results identified KLF4-expressing cells as FLS (Figure 1C and Figure S4 in Supplementary Material).

### Deletion of KLF4 Reduces Autoimmune-Derived Inflammation in a CAIA Mouse Model

We next examined the severity of arthritis *in vivo* by altering KLF4 expression. To do this, we generated RGEN-mediated KLF4 knockout mice on a C57BL/6 background. Clusters of regularly interspaced palindromic repeat (CRISPR)/CRISPR-associated protein 9 was used to delete 13 nucleotides from the KLF4 exon 2 region (Figure S1A in Supplementary Material). Deletion was confirmed by PCR of genomic DNA (Figure S1C in Supplementary Material). The resulting frame-shift mutation in the DNA sequence led to a marked alteration in the amino acid codons of KLF4 proteins (Figure S1B in Supplementary Material). The altered KLF4 protein was not detected by a KLF4 antibody (Figure S1D in Supplementary Material). A previous study showed that the skin barrier function of KLF4 knockout mice generated by homologous recombination is compromised (8). However, RGEN-mediated KLF4 knockout mice did not show an abnormal skin phenotype. Therefore, we checked transcription of a representative skin protection gene, sprr2a, and found that there was no significant difference in sprr2a expression between wild-type and KLF4 knockout mice (Figure S1E in Supplementary Material). The latter mouse strain was named KLF4^Rg−/−^ to distinguish it from KLF4^−/−^ generated by homologous recombination. Detailed information about immunophenotyping of KLF4 mice is presented in Table S1 in Supplementary Material.

**Figure 2:**
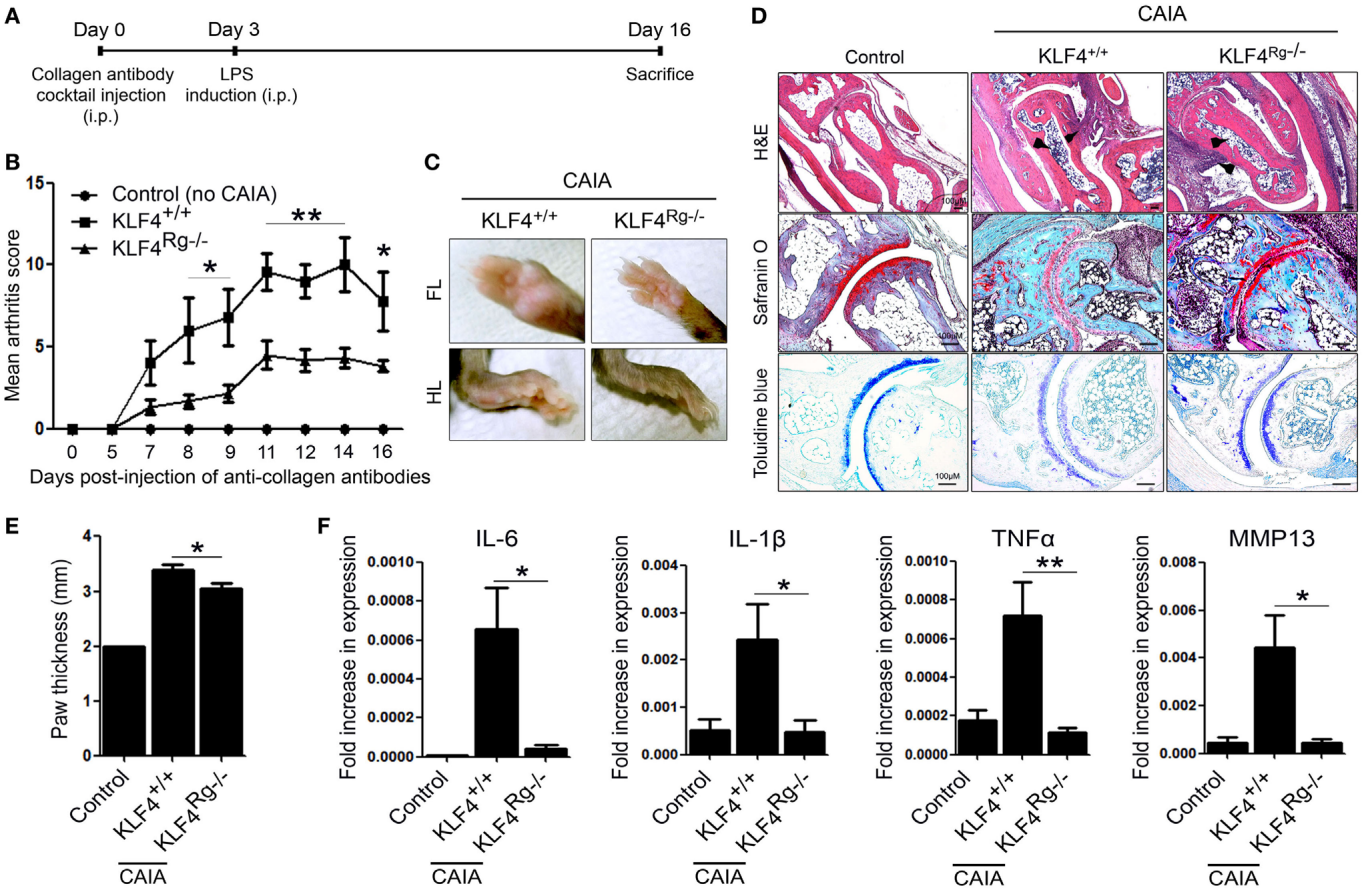
The severity of autoimmune-derived inflammation of Kruppel-like factor (KLF) 4 knockout mice is relieved in a collagen antibody-induced arthritis (CAIA) mouse model. **(A)** Experimental protocol for CAIA induction. **(B)** Mean arthritis score for control mice (not treated with anti-collagen antibodies) (*n* = 3) and for KLF4^+/+^ (*n* = 5) and KLF4^Rg−/−^ (*n* = 6) mice (treated with anti-collagen antibodies). **(C)** Forelimbs (FL) and hindlimbs (HLs) of collagen antibody-treated KLF4^+/+^ and KLF4^Rg−/−^ mice. **(D,E)** Immunohistochemical staining and paw thickness of HL from control, KLF4^+/+^, and KLF4^Rg−/−^ mice. Tissues were stained with hematoxylin and eosin (H&E), Safranin O, and Toluidine blue. Cellular infiltration is pointed by arrow heads. Data are representative of at least five replicates of the staining per each group. Original magnification: H&E, 50×; Safranin O and Toluidine blue, 100×. **(F)** Expression of mRNA encoding IL-6, interleukin (IL)-1β, tumor necrosis factor-alpha (TNF-α), and matrix metallopeptidase 13 (MMP13) in paw cells. Values in panels **(B,E,F)** are expressed as the mean ± SEM (*P < 0.05, **P < 0.01, and ***P < 0.001).

Arthritis was induced by injecting mice with a collagen antibody cocktail on day 0, followed by injection of LPS on day 3. The severity of arthritis in wild-type (KLF4^+/+^) and knockout mice (KLF4^Rg−/−^) was scored, and the data are presented in Figures 2A,B. Mean arthritis score of KLF4^Rg−/−^ mice was much lower than that of KLF4^+/+^ mice at day 8 to day 16 after collagen antibody injection (Figure 2B). This result was supported by examination of the forelimb (FL) and HL (Figure 2C). To investigate joint destruction in more detail, joint tissues from control mice (no collagen antibody treatment) and from KLF4^+/+^ and KLF4^Rg−/−^ mice (treated with collagen antibodies) were subjected to IHC staining. Deletion of KLF4 reduced cell infiltration and cartilage damage in the joints (Figure 2D). In addition, HL paw of KLF4^Rg−/−^ mice was less thicker than HL paw of KLF4^+/+^ mice (Figure 2E). Transcription of IL-6, IL-1, TNF-α, and MMP13 in synoviocytes from the paws of KLF4^Rg−/−^ mice was much lower than that in control or KLF4^+/+^ mice (Figure 2G). These findings suggest that deletion of KLF4 reduces inflammation in a CAIA mouse model.

### KLF4 Overexpression Through Introduction of Minicircle Vectors Results in Severe Autoimmune Arthritis in the CIA Mouse Model

**Figure 3:**
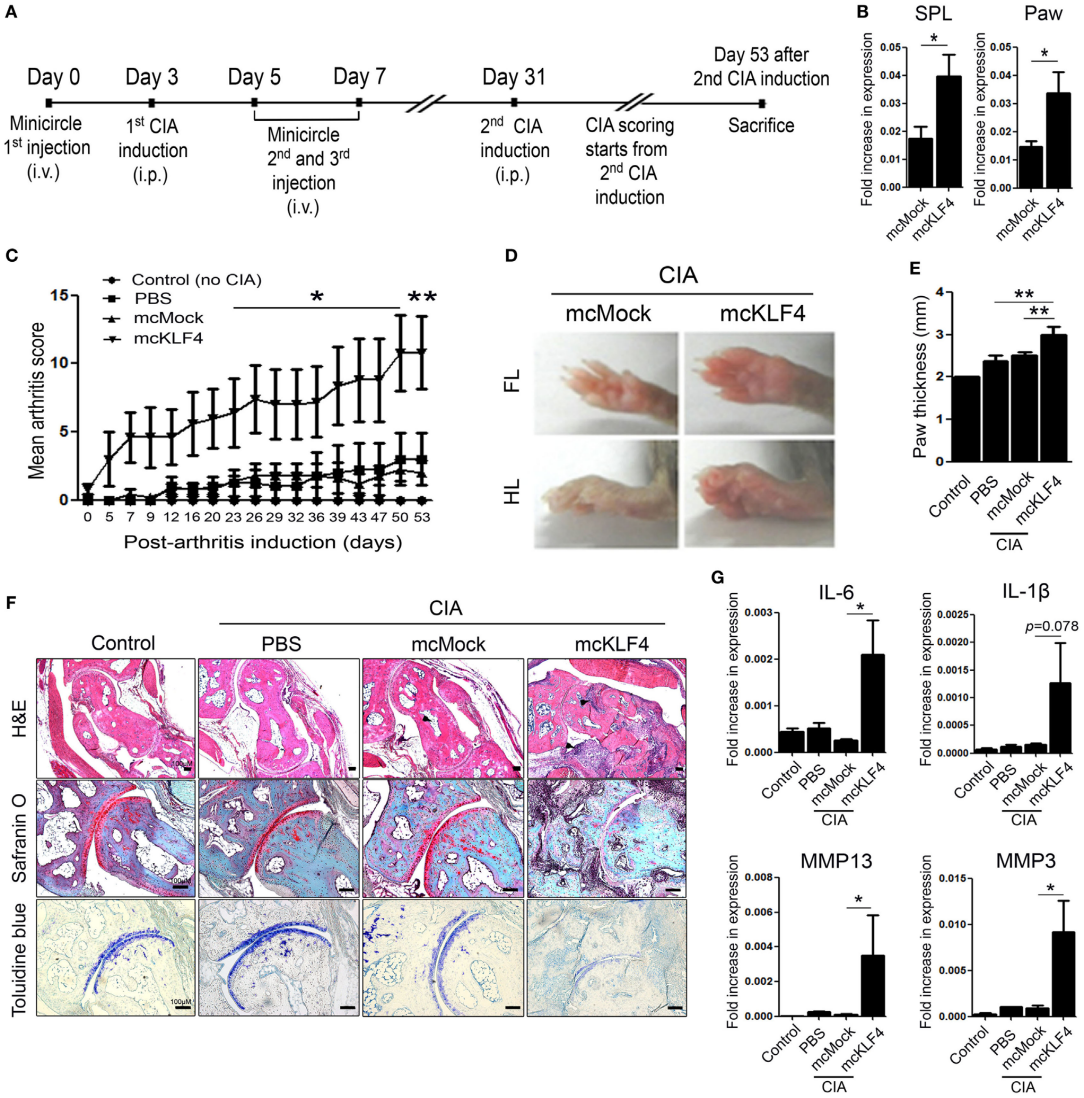
Overexpression of Kruppel-like factor (KLF) 4 in collagen-induced arthritis (CIA) mice results in severe autoimmune arthritis. **(A)** Experimental induction of CIA by intravenous (i.v.) injection of minicircle vectors. **(B)** Expression of KLF4 mRNA in splenocytes (SPL) and paw cells from minicircle Mock vector (mcMock)- and minicircle KLF4 vector (mcKLF4)-injected mouse (*n* = 3). **(C)** Mean arthritis scores for control mice (not induced by collagen) (*n* = 4), for mice induced by collagen and then i.v. injected with phosphate-buffered saline (PBS) (*n* = 4), for mcMock-transduced mice (*n* = 5), and for mcKLF4-transduced mice (*n* = 5). **(D)** Hindlimbs (HLs) and paws of mcMock- and mcKLF4-injected CIA model mice. **(E,F)** Paw thickness and immunohistochemical staining of HLs from control and PBS-, mcMock-, and mcKLF4-injected mice. Tissues were stained as described in Figure 2E. Cellular infiltration is pointed by arrow heads. Data are representative of at least five replicates of the staining per each group. Original magnification: hematoxylin and eosin (H&E), 50×; Safranin O and Toluidine blue, 100×. **(G)** Expression of mRNA encoding interleukin (IL)-6, IL-1β, matrix metallopeptidase 13 (MMP13), or MMP3 in paw cells. Values in panels **(B,C,E,G)** are expressed as the mean ± SEM (*P < 0.05 and **P < 0.01).

Next, we used minicircle vectors to overexpress KLF4 protein in DBA1/J mice. Because parts of the bacterial backbone not necessary for overexpression of the inserted gene are removed by arabinose treatment, minicircle vectors are considered to be suitable plasmids for transgene expression (22). Insertion of the KLF4 open reading frame sequences was verified by treatment with restriction enzymes (Figure S2 in Supplementary Material). The CIA model included phosphate-buffered saline (PBS) treated, mock-treated (mcMock) and transduced with a KLF4 minicircle vector (mcKLF4) groups in which respective reagents were injected three times near the first CIA induction (Figure 3A). After confirming transcription of KLF4 in SPL and paw cells *in vivo* (Figure 3B), we scored the severity of arthritis in the PBS-, mcMock-, and mcKLF4-injected groups. Compared with those of the mcMock-injected group, the mean severity scores of the mcKLF4-injected group began to decrease on day 23 after the second CIA induction (Figure 3C). Both the FL and HL of mcKLF4 mice were more swollen (Figure 3D). The HL paw of mcKLF4 mice was thicker than that of mcMock mice after CIA was induced (Figure 3E). IHC staining revealed that cell infiltration and cartilage damage in the mcKLF4 group was more severe than that in the mcMock group (Figure 3F). Transcription of IL-6, MMP13, and MMP3 was greater in synoviocytes from the paws of mcKLF4-injected mice (Figure 3G). Opposite to the result in KLF4^Rg−/−^ mice, overexpression of KLF4 resulted in severe autoimmune arthritis, accompanied by upregulation of RA-associated inflammatory factors.

### Downregulation of KLF4 With Kenpaullone Alleviates Autoimmune Arthritis in the CIA Mouse Model

**Figure 4:**
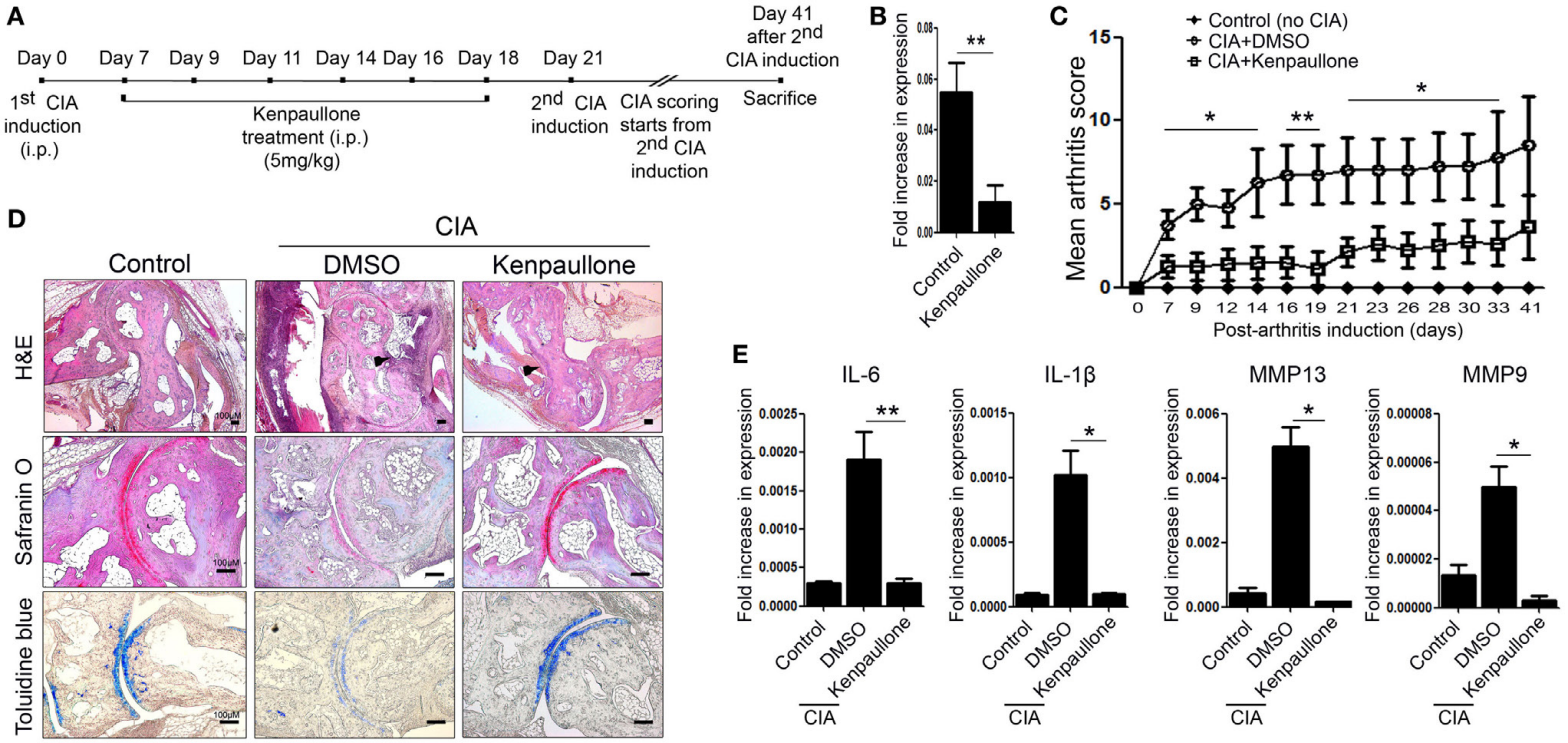
Inhibition of Kruppel-like factor (KLF) 4 with Kenpaullone reduces autoimmune arthritis in the collagen-induced arthritis (CIA) mouse model. **(A)** Experimental induction of CIA and Kenpaullone treatment. **(B)** Expression of KLF4 mRNA by control and Kenpaullone-treated murine fibroblast-like synoviocytes. **(C)** Mean arthritis score for control mice (no collagen induction) (*n* = 3), CIA mice treated with 10% dimethyl sulfoxide (DMSO) (*n* = 4), and CIA mice treated with Kenpaullone (*n* = 8). **(D)** Immunohistochemical staining of the hindlimb. Staining was performed as described in Figure 2E. Cellular infiltration is pointed by arrow heads. Data are representative of at least five replicates of the staining per each group. Original magnification: hematoxylin and eosin (H&E), 50×; Safranin O and Toluidine blue, 100×. **(E)** Expression of mRNA encoding interleukin (IL)-6, IL-1β, matrix metallopeptidase 13 (MMP13), or MMP9 in paw cells. Values in panels **(B,C,E)** are expressed as the mean ± SEM (*P < 0.05 and **P < 0.01).

Next, we examined the effect of downregulating KLF4 expression in the CIA mouse model. To do this, DBA1/J mice with CIA were treated with an inhibitor of KLF4, Kenpaullone (23). Mice received six injections of Kenpaullone or 10% dimethyl sulfoxide every other day started at day 7 from the first CIA inductions (Figure 4A). DMSO was used as a control. Kenpaullone-mediated downregulation of KLF4 was confirmed by real-time q-PCR (Figure 4B). Kenpaullone-treated mice showed less severe autoimmune arthritis than DMSO-treated mice (Figure 4C). Next, we examined cell infiltration and cartilage damage by IHC staining (Figure 4D). Transcription of IL-6, IL-1β, MMP13, and MMP9 was also reduced after Kenpaullone treatment, whereas not all genes are downregulated by Kenpaullone. (Figure 4E and Figure S5 in Supplementary Material). The results confirmed that downregulation of KLF4 alleviates autoimmune arthritis in the CIA mouse model.

### KLF4 Modulates Autoimmune Arthritis by Regulating Murine FLS Cell Survival and Inflammatory Responses

**Figure 5:**
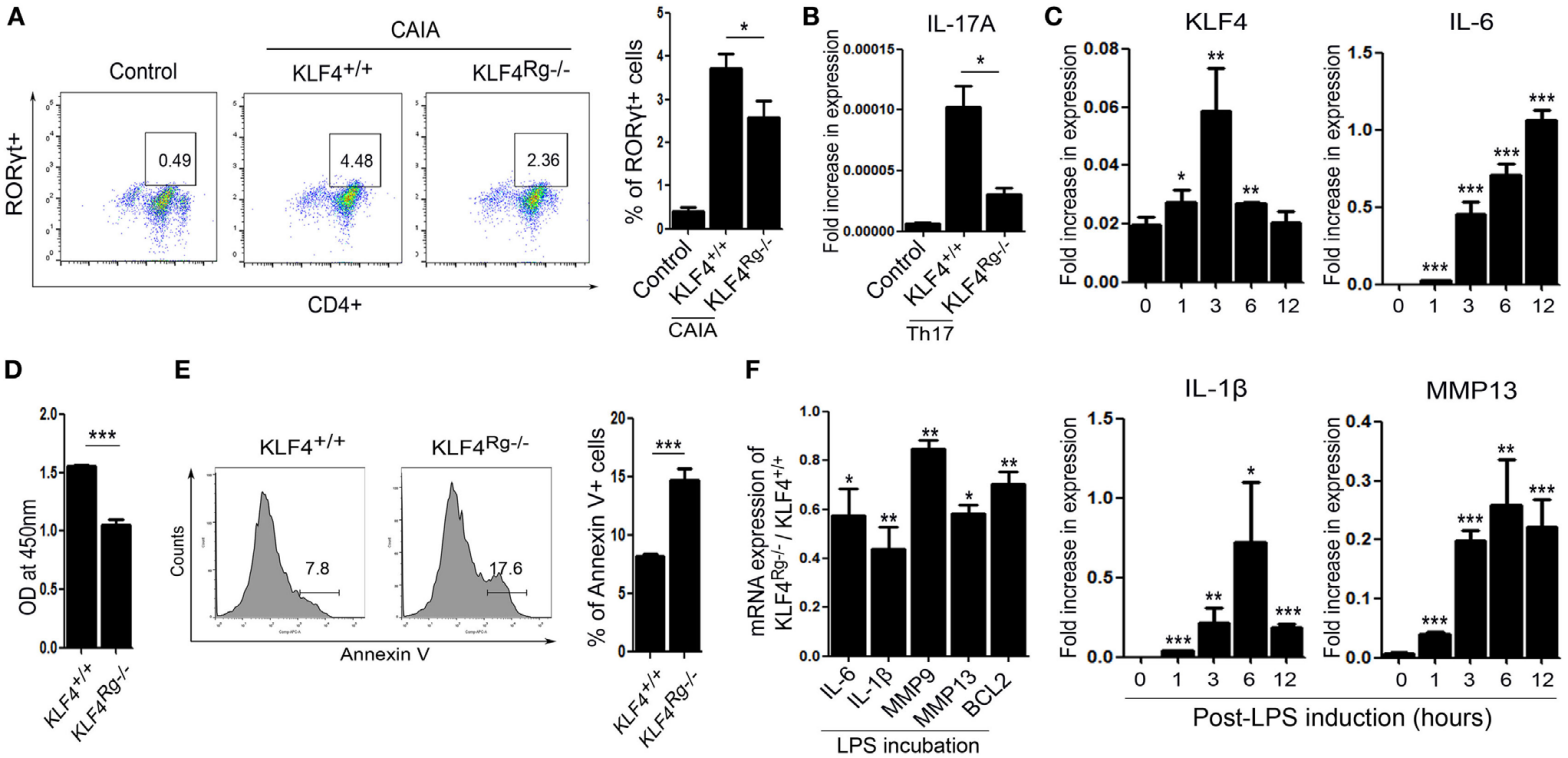
Kruppel-like factor (KLF) 4 affects autoimmune arthritis by regulating fibroblast-like synoviocyte (FLS) cell survival and inflammatory responses *in vitro*. **(A)** Left panel: representative flow cytometry plot showing the percentage of Th17 cells within the murine CD4^+^ T cell population sorted from splenocytes isolated from mice induced with collagen antibody (KLF4^+/+^, KLF4^Rg−/−^) or not (control). Th17 cells were identified as CD4^+^RORγt^+^. Right panel: average percentage of Th17 cells (*n* = 4, control; *n* = 10, KLF4^+/+^ and KLF4^Rg−/−^, each). **(B)** mRNA expression of IL-17A in Th0 (control) and Th17 (KLF4^+/+^, KLF4^Rg−/−^) differentiated CD4^+^ T cells (*n* = 3). **(C)** Expression of mRNA encoding KLF4, interleukin (IL)-6, IL-1β, and matrix metallopeptidase 13 (MMP13) in control and lipopolysaccharide (LPS)-treated murine FLS [as measured by real-time quantitative polymerase chain reaction (q-PCR), *n* = 4]. **(D)** Proliferation of KLF4^+/+^ and KLF4^Rg−/−^ FLS (as measured in a CCK-8 assay). **(E)** Representative flow cytometry plots showing Annexin V-stained FLS from KLF4^+/+^ and KLF4^Rg−/−^ mice (*n* = 4). **(F)** Ratio of mRNA encoding IL-6, IL-1β, MMP9, MMP13, or BCL2 in FLS from KLF4^Rg−/−^ mice to that in FLS from KLF4^+/+^ mice with LPS treat for 24 h (as measured by real-time q-PCR, *n* = 3). Values are expressed as the mean ± SEM (*P < 0.05, **P < 0.01, and ***P < 0.001). Values in panel **(C)** are generated from a comparison between control (0 h) and LPS-treated murine FLS.

We next examined the immune cell subsets involved in autoimmune arthritis regulated by KLF4. Since KLF4 regulates Th17 cell differentiation directly (24), we isolated CD4^+^ T cells and cultured them in Th17 differentiation-inducing medium. Upon induction of CAIA, we noted a reduction in the number of RORγt^+^ T cells (Th17 cells) in KLF4^Rg−/−^ mice Figure 5A and Figure S6 in Supplementary Material. mRNA expression of IL-17A in Th17-differentiated CD4^+^ T cells which were isolated from KLF4^Rg−/−^ SPL was reduced (Figure 5B). Since FLS is one of the main immune-like subsets involved in autoimmune arthritis, we checked the responsiveness of murine FLS to LPS. KLF4 was induced at 3 h post-LPS treatment, other inflammatory factors were highly induced at 3 h later (IL-6, MMP13) or 6 h later (IL-1β) (Figure 5C). Next, we isolated FLS from KLF4^+/+^ and KLF4^Rg−/−^ mice. The result demonstrated that, while proliferation of KLF4^Rg−/−^ FLS was reduced, apoptosis increased *in vitro* assay (Figures 5D,E). Expression of mRNA encoding IL-6, IL-1β, MMP9, MMP13, and the antiapoptotic factor BCL2, was lower in KLF4^Rg−/−^ FLS (Figure 5F). Thus, KLF4 modulates autoimmune arthritis by regulating FLS cell survival and inflammatory responses as well as Th17 differentiation.

### KLF4 Regulates Human FLS Cell Survival and Expression of Inflammatory Factors and MMPs

**Figure 6:**
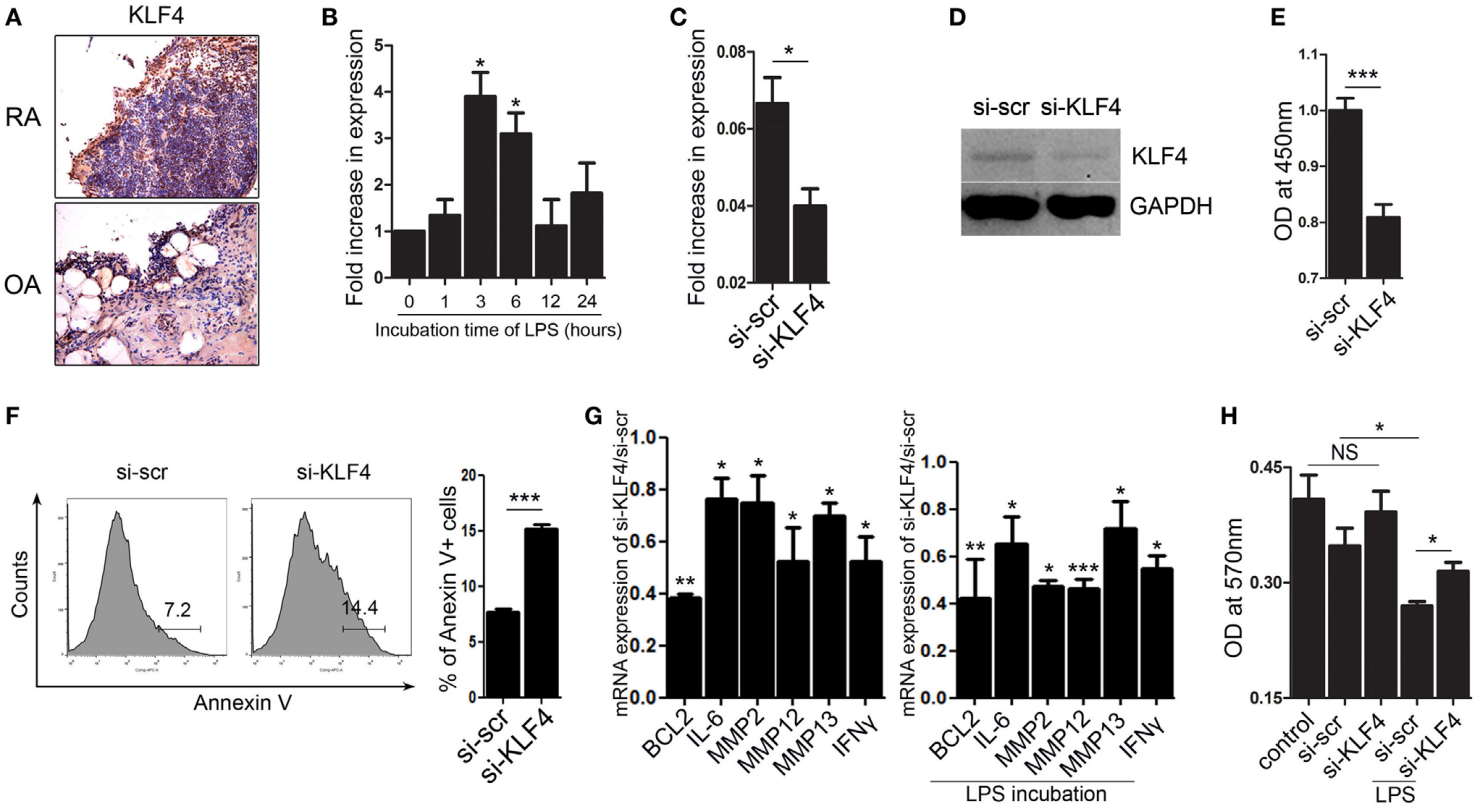
Kruppel-like factor (KLF) 4 regulates human fibroblast-like synoviocyte (FLS) cell survival and expression of inflammatory factors and matrix metalloproteinases. **(A)** Representative images of anti-KLF4 antibody-stained joint tissues from patients with rheumatoid arthritis (RA) and osteoarthritis. **(B)** Expression of mRNA encoding KLF4 in human FLS in response to lipopolysaccharide (LPS) (as measured by qRT-PCR *n* = 3). **(C,D)**, Expression of KLF4 mRNA and protein by FLS transduced with scrambled small-interfering RNA (si-scr) and si-KLF4. **(E)** Proliferation of FLS transduced with si-scr and si-KLF4 (as measured in a CCK-8 assay). **(F)** Representative flow cytometry plots showing Annexin V-stained human FLS transduced with si-scr and si-KLF4 (*n* = 4). **(G)** Left panel: ratio of expression of mRNA encoding BCL2, IFN-γ, MMP2, MMP12, and matrix metallopeptidase 13 (MMP13) in FLS transduced with si-KLF4 to that in FLS transduced by si-scr in the absence of LPS (*n* = 3). Right panel: with LPS treat for 24 h (as determined by real-time quantitative polymerase chain reaction) (*n* = 3). **(H)** Analysis of collagen degradation using culture medium from si-scr- and si-KLF4-transduced FLS treated (or not) with LPS. Control samples were incubated with phosphate-buffered saline. Values are expressed as the mean ± SEM (*P < 0.05, **P < 0.01, and ***P < 0.001). Values in panel **(B)** are generated from a comparison between control (0 h) and LPS-treated human FLS.

Next, we focused on FLS isolated from human joint tissue. We found that synoviocytes from RA patients showed high expression of KLF4 than those from OA patients (Figure 6A and Figure S7 in Supplementary Material). When FLS isolated from RA patients were treated with LPS, we found that KLF4 was induced within 3 h (Figure 6B). Next, we used a lentiviral vector to knock down expression of KLF4 in FLS. Control cells were transduced with si-scr sequence vectors. We found that expression of KLF4 mRNA and protein was downregulated after transduction of si-KLF4 vectors into FLS (Figures 6C,D and Figure S3 in Supplementary Material). Upon knockdown of KLF4, proliferation of FLS decreased, whereas apoptosis increased (Figures 6E,F). Expression of mRNAs encoding BCL2, IL-6, MMP2, MMP12, MMP13, and IFN-γ fell upon KLF4 knockdown in the presence/absence of LPS (Figure 6G). Among the MMP family, MMP13 has the ability to destroy collagen (25); therefore, we evaluated the level of collagen degradation. When treated with LPS, higher degradation of type I collagen was found in FLS (Figure 6H). Among the LPS-treated group, si-KLF4-transduced FLS group showed decreased degradation of type I collagen (Figure 6H). Thus, KLF4 regulates proliferation and apoptosis of FLS, along with expression of inflammatory factors and MMPs.

## Discussion

Here, we used two murine models of autoimmune arthritis to examine the role of KLF4 in disease pathogenesis. The results showed that expression of KLF4 in the synovium of mice with inflammatory arthritis was much higher than that in controls, and that KLF4 plays a major role in disease pathogenesis. KLF4 also regulated FLS proliferation, MMP expression, and secretion of proinflammatory cytokines.

Although the role of KLF4 in the pathogenesis of RA is poorly understood, several studies suggest that it plays a pivotal role in the disease process (12, 23). Since KLF4 not only regulates cellular proliferation but also plays a role in inflammation (7), we hypothesized that KLF4 contributes to development of RA. To the best of our knowledge, this study is the first to report that KLF4 plays a central role in experimental inflammatory arthritis. Using KLF4 knockout mice, we showed for the first time that KLF4 contributes to the pathogenesis of CAIA (Figure 2B). Moreover, overexpression of KLF4 using a minicircle vector induced more severe inflammatory arthritis than overexpression of control minicircle vector in CIA mice (Figure 3C). Taken together, these findings suggest that targeting KLF4 may be a novel method of preventing/treating autoimmune arthritis.

Many mouse models of RA are available; however, care must be taken to select the one most appropriate for the research question. Although a single model of inflammatory arthritis cannot mirror the full complexity of the disease, the use of more than one model may mirror different aspects of the disease (26). Here, we used two murine models of RA (CIA and CAIA), each representing different aspects of RA pathogenesis. CIA is induced by immunization of genetically susceptible strains of mice with heterologous CII emulsified in CFA (27). Following immunization, the animals develop an autoimmune polyarthritis characterized by severe cartilage and bone erosion (28). The CAIA model on the other hand is induced by anti-collagen antibodies (29). Murine CAIA, like CIA, has clinical and immunopathologic features that parallel those in human RA. CAIA induced by autoantibodies targeting components of the cartilage extracellular matrix leads to an inflammatory milieu within the joint, thereby promoting severe arthritis (30). The CAIA model offers several advantages over the CIA model, including rapid disease onset, high uptake rate, and the capacity to use genetically modified mice (e.g., knockouts on a C57BL/6 background) (31). Therefore, we used a KLF4 knockout CAIA model to examine the effect of KLF4 on inflammatory arthritis.

As mentioned earlier, skin phenotype of KLF4 RGEN knockout mice and classical knockout mice using homologous recombination system are different. There are some possibilities to explain differences. First, there are reports that gene expression and cell physiology change in some of the experiments using the PGK-neo knockout method (32, 33). In this regard, it is possible that an incomplete KLF4 transcript could be generated in the classical knockout, which might have interfered with the expression of the skin barrier protein. Second, the possibility of KLF4 off-target effect is less likely, but remains. To expect the possibility of off-target effect of RGEN, both RG1 and RG2 mismatches are counted. Three base pair-mismatched target sequences were found. No other mismatch sequence that is 0–2 bp different from RG1 and RG2 target site sequences was discovered (data not shown). In a recent study, different phenotypes of immunodeficient mouse between RGEN knockout and classical knockout are observed (34). The exact mechanisms for this difference should be clarified through further studies.

The severity of arthritis in KLF4 knockout mice was less than that in wild-type mice (Figures 2A–E). In the CIA model, chemical inhibition of KLF4 reduced the severity of inflammatory arthritis (Figure 4B). KLF4 deficiency attenuated arthritis severity in both CIA and CAIA mice. By contrast, overexpression of KLF4 in CIA mice aggravated arthritis when compared with wild-type CIA mice (Figures 3C–F).

The pathogenesis of RA is enormously complex and involves many cell types. Variable clinical responses to targeted therapies such as TNF blockade, inhibition of T cell co-stimulation, and depletion of B cells demonstrate that the disease is heterogeneous and probably lacks a single mechanism that applies to all patients. FLS within the synovial intimal lining undoubtedly contribute to disease pathogenesis; therefore, targeting this interesting and unique cell type may complement current therapies (13). FLS not only secrete cytokines to activate inflammatory cells but also exhibit a tumor cell-like transformation (14). Proliferation of the FLS population in patients with RA occurs due to an imbalance between cell differentiation, survival, and apoptosis (13). FLS play a key role in RA by producing cytokines that drive inflammation and expression of proteases such as MMPs, which contribute to cartilage destruction and increase invasion of the extracellular matrix, further exacerbating joint damage (35).

Our data show that KLF4 regulates the severity of inflammatory arthritis by acting on FLS. We found that expression of KLF4 in the synovium of CIA mice and RA patients was higher than that in control mice and patients with OA, respectively (Figures 1A and 6A). FLS from the synovium of CIA mice expressed KLF4 (Figure 1B). We also observed that KLF4 regulates proliferation and apoptosis of FLS in both mice and RA patients (Figures 5 and 6). Hyperplasia of the synovial lining in RA is due to disruption of homeostatic mechanisms. As cell division is uncommon in the joint, a lack of synoviocyte apoptosis could cause local accumulation of FLS, although the mechanism responsible for defective apoptosis is not completely understood. *In vitro* culture of FLS derived from RA synovium revealed that KLF4 regulates apoptosis by affecting transcription of the antiapoptotic BCL2 gene (Figures 6F,G). Blockade of KLF4 in FLS from RA patients increased apoptosis and suppressed BCL2 expression. In addition, BCL2 expression was suppressed even more in the presence of LPS (Figure 6G). These findings suggest that KLF4 regulates apoptosis in RA FLS, especially when exposed to an inflammatory milieu.

Matrix metalloproteinases, expressed by FLS, are proteolytic enzymes that degrade the extracellular matrix. Collagenases (MMP1 and MMP13) and stromelysin (MMP3) are particularly important in RA. Here, we showed that expression of MMP13 in the synovium *in vivo* is regulated by KLF4. We used KLF4^Rg−/−^ mice to show that KLF4 regulates expression of the gene encoding MMP13 in the synovium of CAIA mice (Figure 2F). Overexpression of KLF4 led to increased expression of MMP13 in CIA mice (Figure 3G). We also observed that knocking out KLF4 suppressed expression of MMP13 by isolated FLS (Figure 5F). These findings in mice were mirrored by those in FLS from RA patients. Inhibition of KLF4 using siRNA reduced expression of genes encoding MMPs 2, 12, and 13 by FLS from RA patients (Figure 6G, left panel). Furthermore, expression of genes encoding MMPs was suppressed to a greater extent after KLF4 inhibition under conditions of LPS stimulation (Figure 6G, right panel). In particular, knockdown of KLF4 expression in FLS from RA patients reduced collagen degradation by downregulating MMP13 upon LPS stimulation (Figure 6H). Taken together, the results show that KLF4 regulates expression of MMPs by FLS in both mice and RA patients.

Ablation of KLF4 in FLS downregulated expression of mRNA encoding proinflammatory cytokines such as IL-1β and IL-6 (Figure 5F). This is consistent with the results of a recent report examining FLS from RA patients (15). Expression of KLF4 is higher in synovial tissues from RA patients than in those from OA patients; also, KLF4 promotes proinflammatory signaling in FLS (15). Inhibiting KLF4 suppressed expression of the gene encoding IL-6 in both murine FLS and human FLS (Figures 5F and 6G). These data indicate that KLF4 plays a role in the pathogenesis of autoimmune arthritis, and that inhibiting KLF4 attenuates inflammatory arthritis by regulating apoptosis of FLS and their expression of MMPs and proinflammatory cytokines.

Kruppel-like factor 4 is associated with differentiation of Th17 cells, an important contributor to synovitis in RA. KLF4 regulates thymocyte development and proliferation directly, along with IL-17 expression during Th17 differentiation (24). Th17 cells contribute to RA *via* pleiotropic effects, exerted by IL-17 and related cytokines, on osteoclasts, B cells, and monocytes (36). This study showed that knocking out KLF4 reduces CAIA-mediated increases in the number of Th17 cells (Figure 5A). This suggests that inhibiting KLF4 may suppress Th17 differentiation *in vivo*.

In summary, we showed here that KLF4 regulates development of experimental arthritis in CIA and CAIA models. Also, KLF4 regulates proliferation and apoptosis of FLS, along with expression of genes encoding MMPs and proinflammatory cytokines. The results suggest that targeting of KLF4 in FLS might be a novel strategy for RA.

## Ethics Statement

This study was carried out in accordance with the recommendations of Institutional Review Board of Incheon St. Mary’s hospital (license no. OC17TNSI0155). The protocol was approved by the Institutional Review Board of Incheon St. Mary’s hospital. All subjects gave written informed consent in accordance with the Declaration of Helsinki. This study was carried out in accordance with the recommendations of Animal Research Ethics Committee at the Catholic University of Korea. The protocol was approved by the Animal Research Ethics Committee at the Catholic University of Korea.

## Author Contributions

SC, JJ, and KK contributed conception and design of the study and analysis and interpretation of data; SC, KL, HJ, NP, JK, K-HN, and E-KK contributed acquisition of data; SC and K-HN wrote the first draft of the manuscript; SC wrote sections of the manuscript. All the authors contributed to manuscript revision, read and approved the submitted version. JJ and KK had full access to all of the data in the study and take responsibility for the integrity of the data and the accuracy of the data analysis.

## Conflict of Interest Statement

The authors declare that the research was conducted in the absence of any commercial or financial relationships that could be construed as a potential conflict of interest.
