# Supplementary material for: Kruppel-Like Factor 4 Positively Regulates Autoimmune Arthritis in Mouse Models and Rheumatoid Arthritis in Patients via Modulating Cell Survival and Inflammation Factors of Fibroblast-Like Synoviocyte
Source: Front Immunol. 2018 Jun 27;9:1339. doi: 10.3389/fimmu.2018.01339 (PMC6030377; doi:10.3389/fimmu.2018.01339)
Supplement: Supplementary file 1 [file data_sheet_1.docx]

**Mouse Klf4**

**Exon2**

GCAGCCACCTGGCGAGTCTGACATGGCTGTCAGCGACGCTCTGCTCCCGTCCTTCTCCACGTTCGCGTCCGGCCCGGCGGGAAGGGAGAAGACACTGCGTCCAGCAGGTGCCCCGACTAAC

|  |  |  | Mismatches | | | |
| --- | --- | --- | --- | --- | --- | --- |
|  | **RGEN Target** | **Direction** | **0 bp** | **1 bp** | **2 bp** | **3 bp** |
| Ex2_RG1 | TCCTTCTCCACGTTCGCGTCCGG | - | 1 | 0 | 0 | 0 |
| Ex2_RG2 | TCGCGTCCGGCCCGGCGGGAAGG | + | 1 | 0 | 0 | 3 |

This table presents the number of mismatches that have different base pair sequences from RGEN target sequences..

For example, if we focused on RG2 : TCGCGTCCGGCCCGGCGGGAAGG,

3bp = 3 means “ There are 3 target sites that target sequences are 3 base pair different from RG2 target site sequence.”

“ lower case means mismatch sequence on table below”

| Target site | Location | Target site | direction |
| --- | --- | --- | --- |
| TCGCGTCCGGCCCGGCGGGANGG | Chr1 | TCGaGTCCGagCCGGCGGGAGGG | + |
| TCGCGTCCGGCCCGGCGGGANGG | Chr2 | cCGCGTCaGGCCCGGCcGGAGGG | + |
| TCGCGTCCGGCCCGGCGGGANGG | Chr11 | cCGCGTCCGGgCCGGCGGtATGG | + |
