# Supplementary material for: Kruppel-Like Factor 4 Positively Regulates Autoimmune Arthritis in Mouse Models and Rheumatoid Arthritis in Patients via Modulating Cell Survival and Inflammation Factors of Fibroblast-Like Synoviocyte
Source: Front Immunol. 2018 Jun 27;9:1339. doi: 10.3389/fimmu.2018.01339 (PMC6030377; doi:10.3389/fimmu.2018.01339)
Supplement: Supplementary file 5 [file image_2.PDF]

Supplementary Material

Kruppel-like Factor 4 Positively Regulates Autoimmune Arthritis in Mouse Models and Rheumatoid Arthritis in Patients via Modulating Cell Survival and Inflammation Factors of Fibroblast-like Synoviocyte

Seungjin Choi*, Kijun Lee, Hyerin Jung, Narae Park, Jaewoo Kang, Ki-Hoan Nam, Eun-Kyeong Kim, Ji Hyeon Ju, MD and Kwi Young Kang

*** Correspondence:** Ji Hyeon Ju, MD, PhD: juji@catholic.ac.kr

and Kwi Young Kang, MD, PhD : kykang@catholic.ac.kr

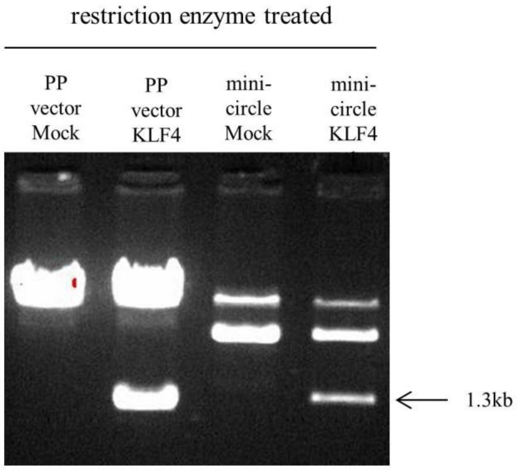

Supplementary Figure 2. Restriction enzyme digestion of the minicircle vector. Insertion of the KLF4 open reading frame (ORF) of the Parental vector (PP) and minicircle KLF4 vector is shown on 1.3kbp cleaved by BamH1 and Xho1. *PP: parental plasmid
