# Supplementary material for: Kruppel-Like Factor 4 Positively Regulates Autoimmune Arthritis in Mouse Models and Rheumatoid Arthritis in Patients via Modulating Cell Survival and Inflammation Factors of Fibroblast-Like Synoviocyte
Source: Front Immunol. 2018 Jun 27;9:1339. doi: 10.3389/fimmu.2018.01339 (PMC6030377; doi:10.3389/fimmu.2018.01339)
Supplement: Supplementary file 8 [file image_5.PDF]

Supplementary Material

Kruppel-like Factor 4 Positively Regulates Autoimmune Arthritis in Mouse Models and Rheumatoid Arthritis in Patients via Modulating Cell Survival and Inflammation Factors of Fibroblast-like Synoviocyte

Seungjin Choi*, Kijun Lee, Hyerin Jung, Narae Park, Jaewoo Kang, Ki-Hoan Nam, Eun-Kyeong Kim, Ji Hyeon Ju, MD and Kwi Young Kang

*** Correspondence:** Ji Hyeon Ju, MD, PhD: juji@catholic.ac.kr

and Kwi Young Kang, MD, PhD : kykang@catholic.ac.kr

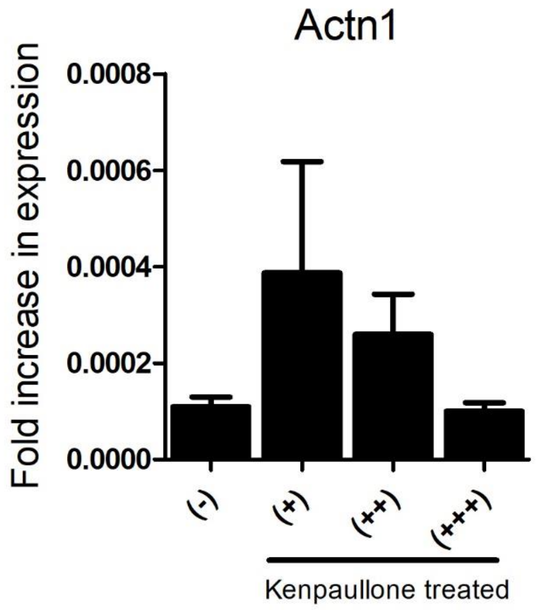

Supplementary Figure 5. Expression of Actn1 mRNA on Kenpaullone-treated human fibroblast-like synoviocytes.
